# Urinary Tract Infections in Kidney Transplant Recipients—Is There a Need for Antibiotic Stewardship?

**DOI:** 10.3390/jcm11010226

**Published:** 2021-12-31

**Authors:** Jens Strohaeker, Victoria Aschke, Alfred Koenigsrainer, Silvio Nadalin, Robert Bachmann

**Affiliations:** Department of General, Visceral and Transplantation Surgery, University Hospital of Tuebingen, 72076 Tuebingen, Germany; Jens.Strohaeker@med.uni-tuebingen.de (J.S.); Victoria.Aschke@med.uni-tuebingen.de (V.A.); Alfred.Koenigsrainer@med.uni-tuebingen.de (A.K.); Silvio.Nadalin@med.uni-tuebingen.de (S.N.)

**Keywords:** urinary tract infection, kidney transplantation, asymptomatic bacteriuria, fluoroquinolones, antibiotic stewardship

## Abstract

(1) Background: Urinary tract infections (UTI) are the most common infections after kidney transplantation. Given the risk of urosepsis and the potential threat to the graft, the threshold for treating UTI and asymptomatic bacteriuria with broad spectrum antibiotics is low. Historically fluoroquinolones were prescription favorites for patients that underwent kidney transplantation (KT). After the recent recommendation to avoid them in these patients, however, alternative treatment strategies need to be investigated (2) Methods: We retrospectively analyzed the charts of 207 consecutive adult kidney transplantations that were performed at the department of General, Visceral and Transplantation Surgery of the University Hospital of Tuebingen between January 2015 and August 2020. All charts were screened for the diagnosis and treatment of asymptomatic bacteriuria (ASB) and urinary tract infections (UTI) and the patients’ clinical characteristics and outcomes were evaluated. (3) Results: Of the 207 patients, 68 patients suffered from urinary tract infections. Patients who developed UTI had worse graft function at discharge (*p* = 0.024) and at the 12 months follow-up (*p* < 0.001). The most commonly prescribed antibiotics were Ciprofloxacin and Piperacillin/Tazobactam. To both, bacterial resistance was more common in the study cohort than in the control group. (4) Conclusions: Urinary tract infections appear to be linked to worse graft functions. Thus, prevention and treatment should be accompanied by antibiotic stewardship teams.

## 1. Introduction

Urinary tract infections (UTI) are the most common infection in the early postoperative phase after kidney transplantation (KT) [1,2]. The incidence of a UTI ranges from 4–80% [3,4,5]. UTIs are defined as the growth of 10^5^ colony forming units on a proper urine sample (i.e., morning urine, puncture urine or from sterile single catheterization) in the presence of symptoms such as dysuria, urinary frequency or localized pain [6]. In contrast to UTIs asymptomatic bacteriuria (ASB) is defined as the presence of ≥10^5^ colony-forming units of bacteria in the absence of symptoms [7]. Asymptomatic bacteriuria is encountered in around 25% of KT recipients [8]. Thus, ASB is commonly screened for and treated in the early posttransplant phase [9], even though there is ongoing controversy about the impact of ASB and UTI on the overall patient and graft outcome [10,11,12]. Several studies have put infections in the context of limited long-term graft function [13,14,15]. In transplant recipients the threshold for treatment is commonly set lower due to the increased risk of septicemia or atypical presentation due to immunosuppression. The incidence of UTI is assumed to be lower in living donation kidney transplantation since these patients often have shorter waiting time, higher volume urine output prior to and during transplantation as well as early onset graft function [16].

A perioperative antibiotic prophylaxis is standard in KT. Overall very heterogeneous regimens exist [17]. Since 2018 the European Association of Urology Guidelines recommend a single shot antibiotic prophylaxis based on a multicentric randomized trial [18,19]. The most common pathogens found in urine samples after KT are *Escherichia coli Enterococcus faecalis* and *Klebsiella pneumoniae* [6,17]. Gram-negative bacteria are usually more pathogenic than gram-positive *Enterococci*. Fluoroquinolones were among the most common antibiotics in the treatment of urinary tract infections in KT recipients given their widespread availability, intravenous and peroral formulations and excellent penetration into the urinary tract [20,21]. On top of that fluoroquinolones offer decent coverage of most uropathogenic bacteria, including *Pseudomonas species* [22,23]. After the FDA warning in 2019 regarding the use of fluoroquinolones both in general and KT recipients in particular, clinicians were forced to reevaluate their empiric antibiotic choice of antibiotics in these patients [24].

For the purpose of this study we evaluated our antibiotic prescription practice during the first 30 days after kidney transplant, the incidence of ASB and UTIs and compared the antibiotic susceptibility of the isolated urinary tract bacteria from KT recipients to those isolated from the local population based on data provided by our hygiene department.

## 2. Materials and Methods

### 2.1. Data Acquisition

We retrospectively screened our hospital information system for all patients who underwent kidney transplantation at the department of General, Visceral and Transplantation Surgery of the University Hospital of Tuebingen, Germany. All adult patients (age ≥ 16 years) who underwent KT at our center between January 2015 and August 2020 were included in the final analysis. Patients that underwent simultaneous pancreas or liver transplantation were excluded. The medical reports of these patients were screened for intra- and perioperative microbiological cultures within 30 days after transplantation. Urinary cultures were sent at the surgeon’s discretion as well as routinely every Monday until discharge. The control group consisted of all urinary tract bacterial isolates cultured by the department of microbiology and hygiene from both outpatients and inpatients. Duplicate isolates were removed.

The study was performed on a consecutive database and was approved by the local ethics committee.

### 2.2. Immunosuppressive Protocol

Induction therapy was 500 mg of iv Methylprednisolone as well as either Basiliximab (20 mg/day on Day 0 and Day 4) for low immunologic risk or Anti-Thymocyte Globulin (1.5 mg/kg/day on Day 0 Day 1 Day 2) for intermediate risk or an Anti-CD56-Antibody (Alemtuzumab 20 mg) for high risk patients (without mycofenolic acid until lymphocytes have regenerated). Maintenance immunosuppression consisted of steroid tapering, calcineurin inhibitor (usually tacrolimus 0.1 mg/kg/day starting day 1) as well as mycofenolic acid (1 g Q12h).

### 2.3. Perioperative Antibiotic Prophylaxis

Perioperative antibiotics consisted either of a Single-Dose Beta-Lactam (Ampicillin/Sulbactam or Cefotaxime) or 3 × 3 g of Ampicillin/Sulbactam (Q0h/12h/24h) at the transplant surgeons’ discretion. Prior to 2018 patients with a leukocyte depleting induction therapy received Trimethoprim-Sulfametoxazole (TMP-SMX) 960 mg 3×/week for Pneumocystis prophylaxis. From 2018 all patients received Pneumocystis prophylaxis with TMP-SMX 960 mg 3× per week. Prior to ureteral anastomosis the bladder was irrigated with Gentamicin.

### 2.4. Treatment Protocols for Urinary Tract Infections

Per protocol all episodes of urinary tract infections and asymptomatic bacteriuria were supposed to be treated with antibiotics. When clinical suspicion for a UTI arose (abnormal urinary dipstick with typical symptoms of UTI or systemic signs of inflammation) empiric antibiotics were initiated. Those were terminated if urinary cultures were negative or a different reason for symptoms/inflammation was detected. Prior to the recommendation to avoid fluoroquinolones the suggested empiric treatment was Ciprofloxacin with a dose equivalent of 250 mg twice daily for 5 days with dose adjustments according to the glomerular filtration rate (GFR). After the recommendation to avoid fluoroquinolones the recommended empiric antibiotic was Piperacillin/Tazobactam 4500 mg 3×/day for 5 days with dose adjustments according to GFR. For critically ill patients Meropenem 1000 mg 3×/day was the recommended substance and dose.

Whenever possible deescalation of treatment according to urinary culture results and antibiotic resistance testing was recommended.

### 2.5. Urinary Catheter and Double-J-Stent Management

Urinary catheters were placed preoperatively and removed on postoperative day (POD) 5 in patients with retained diuresis prior to transplant. In patients with reduced urine output prior to transplantation the urinary catheter was scheduled to be removed on POD. Ureterocystoneostomy was performed according to Lich-Gregoir. Intraoperatively a Double-J-ureteral stent was placed which was removed on POD 21 per protocol (or earlier if urinary tract infection was detected).

### 2.6. Postoperative Follow-Up

Until discharge all patients received blood workup three times per week (per protocol) as well as routine urinary sampling (chemistry and culture) every Monday or whenever clinical or laboratory signs of inflammation/infection were present. All patients had at least two ultrasounds per week to evaluate graft perfusion, rule out ureteral stenosis and perirenal fluid collections. Renal biopsies were taken on indication and assessed according to the Banff classification. Approximately one week after discharge all patients were seen at the outpatient clinic for blood and urine workup, clinical and sonographic follow-up.

### 2.7. Clinical Definitions

Urinary tract infections were defined according to the guidelines of the Center of Disease Control. Asymptomatic patients without systemic signs of infection and >10^5^ colony forming units on a proper urinary sample were considered to have asymptomatic bacteriuria. When symptoms were present the diagnosis of an uncomplicated UTI was made unless signs of septicemia were present. In ten patients we found clinical symptoms in combination with pathologic urinary findings (nitrite and/or leukocyturia) in combination with systemic signs of inflammation but were unable to culture a pathogen. We still recorded these patients as urinary tract infections.

Multi-resistant gram-negative bacteria were defined as follows: when bacteria were resistant to three out of four of the following substance classes (acylureidopenicillin, 3rd generation cephalosporins, fluoroquinolones, carbapenems) they were classified as 3-MRGN. Bacterial strains that were resistant to all four of the aforementioned substance classes were classified as 4-MRGN.

### 2.8. Microbiological Culture, Identification of Strains and Resistance Testing

Urinary samples were sent to the microbiology department for urinary culture every Monday and once there was suspicion for urinary tract infections. Bacteria were cultured on standard columbia sheep blood agar (Oxoid, Wesel, Germany), Cystine Lactose Electrolyte Deficient (CLED)-agar (self-made by the department of microbiology) and Colistine-Nalidixic Acid (CNA)-agar (BioMerieux, Marcy-l’Etoile, France). Identification of strains was done using matrix-assisted laser desorption/ionization time-of-flight mass spectrometry (MALDI-TOF) (Bruker, Billerica, MA, USA). Antimicrobial resistance was defined according to European Committee on Antimicrobial Susceptibility Testing (EUCAST) thresholds and minimal inhibitory concentrations were measured with a VITEK^®^ 2 (BioMerieux, Marcy-l’Etoile, France)

### 2.9. Statistics

Comparison between groups was carried out by the Chi-Square test (Χ²) or Fisher’s exact test (FET) for nominal variables and the Mann–Whitney U-test (MWU) for continuous variables, as appropriate. A probability of less than 0.05 was considered to be statistically significant. All *p*-values reported were the result of 2-sided testing. Where needed, Bonferroni correction was applied. Statistical analysis was carried out using IBM SPSS Statistics for Windows, Version 26.0 (IBM Corp., Armonk, NY, USA).

## 3. Results

### 3.1. Clinical Characteristics

We analyzed the charts of all consecutive adult (age ≥ 16 years) kidney transplant recipients that have undergone KT at the department of General, Visceral and Transplantation Surgery of the University Hospital Tuebingen, Tuebingen, Germany from January 2015 to August 2020. Per protocol all KT recipients had urine samples sent for culture every Monday of their initial stay as well as at the surgeons’ discretion based on postoperative labs and clinical course.

The median length of stay was 20 days (range 10–53).

During the study period, 207 patients met the inclusion criteria. Of these 207 patients 73 patients underwent living donation kidney transplantation (35%) 134 received a kidney from a deceased donor (65%). Overall 119 (57%) of patients were male compared to 88 (43%) females. The median age during KT was 55 years with a standard deviation (SD) of ±14 years. During the first 30 days after their transplant 130 patients (63%) were suspected to have a urinary tract infection. Clinical suspicion was raised when symptoms of a UTI were present and urinary dipstick showed leukocyturia or nitrite. Of these 130 patients 68 patients fulfilled the criteria of a UTI (typical symptoms and/or clinical/laboratory signs of inflammation) In 58 of these 68 patients growth of a urinary pathogen on urine culture was recorded. In ten patients we found typical symptoms accompanied by laboratory and urine chemistry findings consistent with UTI (e.g., nitrite, increase of c-reactive protein, leukocytosis) but were unable to culture the pathogen. For details, see Table 1.

### 3.2. Microbiological Results

The most common isolates from urinary cultures were gram-negative enterobacteriaceae as well as enterococcus species. *E. coli* was the leading pathogen and was found in 49 cultures followed by *E. faecium* (*n* = 23) and *E. faecalis* (*n* = 23). From 68 cultures drawn during UTI 73 uropathogenic bacteria were cultured. Mixed cultures of two uropathogenic cultures were recorded in 5 cultures. One patient developed four individual episodes of urinary tract infections and grew four different bacteria (*E. coli*, Vancomycin sensitive *E. faecium*, Vancomycin-resistant *E. faecium* followed by a different strain of *E. coli*). Fifty-nine individual episodes of ASB were recorded. A total of 62 uropathogenic bacteria were cultured during ASBs. For details see Table 2.

The culture results during urinary tract infections compared to asymptomatic bacteriuria is distinctly different. In total, 135 bacterial isolates were cultured. Of these, 88 (65%) were gram-negative and 46 (34%) were gram-positive. *Ureaplasma* (1%) was excluded from this analysis due to its inability to stain on gram stains. Additionally, five cultures yielded two uropathogenic bacteria in relevant colony forming units. Given that we cannot determine their individual impact on the infection these bacteria are considered individual entities for the following analyses. Of the 88 gram-negative bacteria 56 were recorded during a UTI (64%) compared to 32 (36%) isolates that were found in ASB. In contrast, 30 out of 46 (65%) of gram-positive isolates were found to be asymptomatic bacteriuria and only 16 (35%) gram-positive bacteria were recorded during an episode of urinary tract infections (*p* = 0.001).

### 3.3. Antibiotic Prescription

Of the 207 kidney transplant recipients 166 (80%) underwent at least a single course of antibiotic treatment (excluding perioperative and periinterventional prophylaxis). The median duration of antibiotic treatment was 5 days with a SD of ±4 days. Overall, 262 courses of antibiotic treatments were prescribed to the patient cohort within the first 30 days post transplant. The most common indication for antibiotic treatment was (suspected) urinary tract infection. Five patients in our cohort were treated for urosepsis (2.4%). One of these patients was treated for an uncomplicated UTI prior to the development of urosepsis and one was treated for an episode of uncomplicated UTI after successful treatment of a urosepsis. Excluding the three patients that were treated for urosepsis as their only urinary tract infection a total of 127 patients were treated for suspected UTI. Overall, 180 courses of antibiotics were prescribed to these 127 patients. Of these 127 patients, 85 patients received a single course of antibiotics, 33 patients received two courses, 7 received three courses and two patients received four courses of antibiotics. Patients that underwent at least a single course of antibiotics during their initial stay had significantly worse GFR at discharge (Mean 49.4 mL/min ± 17 vs. 39.7 mL/min ± 19; *p* = 0.024), 3 month follow-up (53.6 mL/min ± 18 vs. 44.8 mL/min ±18; *p* = 0.002) and 12 month follow-up (53.4 mL/min ± 15 vs. 46.4 mL/min ± 19; *p* = 0.009). Approximately 10% of patients received dosages that were above the commended doses. In contrast in about 25% of these patients a rise in creatinine was documented.

Of the 180 suspected UTIs, fluoroquinolones were the most commonly prescribed antibiotic (Ciprofloxacin *n* = 101, Levofloxacin *n* = 1) followed by Piperacillin/Tazobactam (*n* = 17), aminopenicillins (Ampicillin/Sulbactam *n* = 6; Amoxicillin/Clavulanic acid *n* = 6), Meropenem (*n* = 8), Trimethoprim-Sulfamethoxazole (*n* = 5), cephalosporins (Cefuroxime *n* = 4, Cefpodoxime *n* = 1, Ceftriaxone *n* = 1), Fosfomycin (*n* = 4), Pivmecillinam (*n* = 1) Nitrofurantoin (*n* = 1), Linezolid (*n* = 17) and others (*n* = 7).

### 3.4. Antibiotic Resistance

Overall nine patients presented with multiresistant gram-negative bacteria (MRGN). All nine were 3-mrgn (*E. coli n* = 7, *Enterobacter cloacae n* = 1, *Citrobacter koseri*, *n* = 1). Of the gram-negative bacteria a relevant percentage was resistant to Piperacillin/Tazobactam, 3rd generation cephalosporins and Ciprofloxacin. None of our patients had carbapenem-resistant gram-negative bacteria. Ciprofloxacin was by far the most prescribed antibiotic in our department. While there were only a few Ciprofloxacin-resistant non-coli gram-negative bacteria, *E. coli*—the most common isolate—was resistant to fluoroquinolones in 45%.

**Figure 1:**
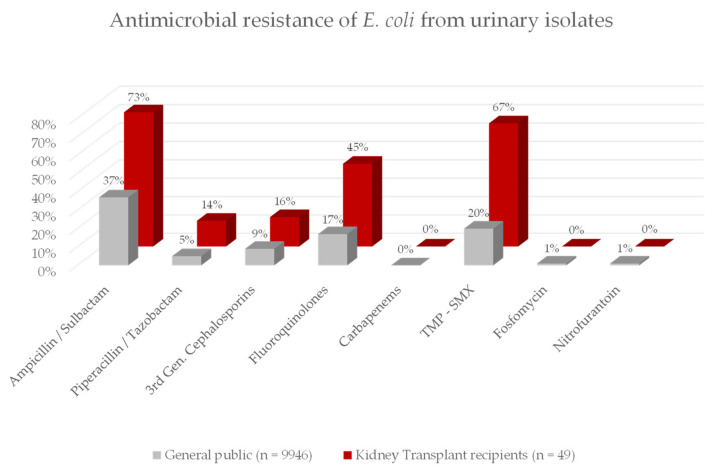
This figure shows the antimicrobial resistance to commonly used antibiotics of *E. coli* in our Kidney Transplant (KT) cohort compared to the local general public. Resistance to Ampicillin / Sulbactam, fluoroquinolones and Trimethoprime-Sulfamethoxazole (TMP–SMX) is substantially higher in the Kidney Transplant cohort.

We specifically compared resistance patterns in the bacteria grown from urinary samples of KT recipients and the general population (control group) that had urine sent for urinary culture at the university hospital of Tuebingen. Resistance patterns were distinctly different between the KT recipients and the control group (CG). The most commonly isolated gram-negative bacteria was *E. coli* (*n* = 49 in KT recipients, *n* > 9000 in CG). In KT recipients *E. coli* was resistant to Ampicillin/Sulbactam in 73%, to Piperacillin/Tazobactam in 14%, to 3rd generation cephalosporins in 16%, to fluoroquinolones in 45%, to carbapenems in 0%, to TMP-SMX in 67%, to Fosfomycin in 0% and to Nitrofurantoin in 0%. In the control group *E. coli* was resistant to Ampicillin / Sulbactam in 37%, to Piperacillin/Tazobactam in 5%, to 3rd generation cephalosporins in 9%, to fluoroquinolones in 17%, to carbapenems in 0%, to TMP-SMX in 20%, to Fosfomycin in 1% and to Nitrofurantoin in 1%. For details see Figure 1.

**Figure 2:**
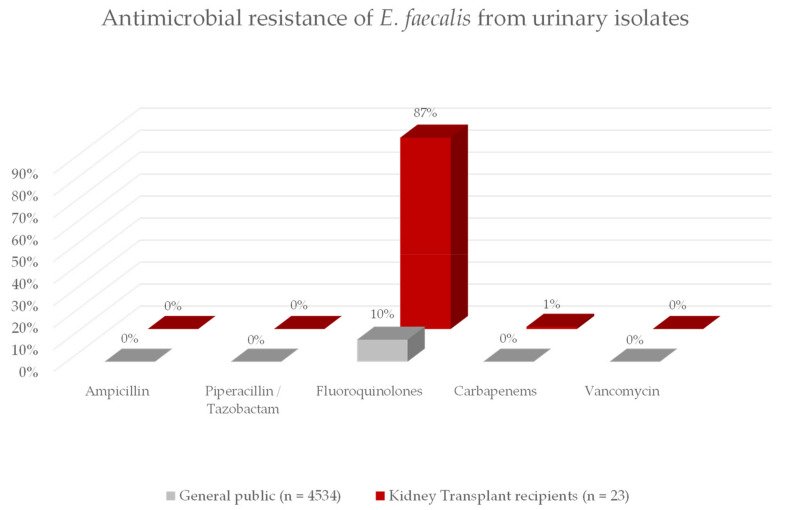
This figure shows the antimicrobial resistance to commonly used antibiotics of *E. faecalis* in our KT cohort compared to the local general public. While the resistance to betalactam antibiotics is comparable, nearly all isolates in the KT cohort are resistant to fluoroquinolones whereas resistance to fluoroquinolones was uncommon in the general public.

The most commonly isolated gram-positive bacteria was *E. faecalis* (*n* = 23 in KT recipients, *n* > 4500 in CG). In KT recipients *E. faecalis* was resistant to Ampicillin/Sulbactam in 0%, to Piperacillin/Tazobactam in 0%, to Fluoroquinolones in 87%, to carbapenems in 4%. In the control group *E. faecalis* was resistant to Ampicillin/Sulbactam in 0%, to Piperacillin/Tazobactam in 0%, to fluoroquinolones in 10%, to carbapenems in 0%. For details see Figure 2.

### 3.5. Bacterial Resistance to Empiric Antibiotic Treatment Options

We calculated the resistance rates all isolated bacteria had to different empiric treatment options. Including both gram-negative and gram-positive bacteria in both UTI and ASB overall bacterial resistance was high. Resistance to Ampicillin/Sulbactam was present in 76 of the 135 strains (56%), to Piperacillin/Tazobactam in 35 strains (26%), to 3rd generation cephalosporins in 62 strains (46%), to fluoroquinolones in 52 strains (39%) and to carbapenems in 23 strains (17%). 

A large proportion of the resistance to Piperacillin/Tazobactam and carbapenems is due to the prevalence of Enterococcus species. Excluding Enterococcus antimicrobial resistance to Ampicillin/Sulbactam was present in 58 bacteria (65%) to Piperacillin/Tazobactam in 13 strains (15%), to 3rd generation cephalosporins in 17 strains (19%), to fluoroquinolones in 26 strains (29%) and to carbapenems in zero bacteria (0%).

## 4. Discussion

Urinary tract infections are the most common infection in the early postoperative phase after kidney transplantation [1,2]. They can lead to sepsis and are a potential threat to life. Aside from progression to septicemia, recent data has suggested a negative impact of even single episodes of (complicated) urinary tract infections on allograft function [13,15,25,26,27,28]. In our cohort urinary tract infections were accompanied by significantly worse creatinine clearance both at the end of our study period and one year after KT. Thus, prevention and adequate treatment of urinary tract infections in KT recipients is crucial. While there is increasing data that asymptomatic bacteriuria (ASB) does not affect graft function [26], inadequate use of antibiotics certainly has the potential to do so [29]. However, the decreased GFR in patients that underwent antibiotic treatment in our cohort should not be interpreted in a vacuum. While about 10% were treated with antibiotic doses that were above the recommended limit more than 25% of patients had a deterioration of graft function. The increasing use of extended donor allocation and overall comorbidities of the recipients adds to this complexity.

Several measures have been proposed to prevent infections in KT recipients. A single shot antibiotic is administered prior to incision [18]. Prolonged antibiotics have not proven beneficiary for the prevention of urinary tract or surgical site infection [17]. In order to decontaminate the bladder we perform intraoperative Gentamicin irrigation of the bladder prior to ureteral anastomosis, which has been proven promising in patients with recurrent UTIs [30]. The presence of indwelling catheters, mainly ureteral stents and Foley catheters is an independent risk factor of urinary tract infections [31]. Our ongoing practice is to remove the ureteral stent 21 days after KT or earlier if there is suspicion for bacteriuria or infection in order to remove biofilms. This approach is backed by Visser et al. who proclaimed that the ideal timing of stent removal is three weeks post-transplant [32].

Several prophylactic antibiotic regimens have been studied. Ciprofloxacin was shown to prevent UTIs 25 years ago, but was later reserved for treatment rather than prophylaxis [33]. Aside from a recent randomized trial that showed the preventative efficacy of Fosfomycin [34] the best studied drug is TMP-SMX. TMP-SMX is recommended for the prevention of *Pneumocystis jirovecii* pneumonia and is usually administered in a prophylactic dose for 6–12 months after KT [3]. The Mayo clinic group reported a reduction in UTIs when TMP-SMX was prescribed for 6 months after KT [6]. Horwedel et al. also described a lower rate of septicemia while patients were on TMP-SMX prophylaxis [35]. In contrast, Singh et al. reported no difference in the incidence of urinary tract infections and asymptomatic bacteriuria in patients that underwent *Pneumocystis* prophylaxis in their cohort [36]. We changed our protocol in 2018 and initiated TMP-SMX prophylaxis in all KT recipients. At our center the UTI rate was significantly lower in patients that were on TMP-SMX.

While it used to be common practice to administer periinterventional antibiotics (usually Ciprofloxacin) for ureteral stent removal [37], Lee et al. reported that the omission of additional periinterventional antibiotics was not disadvantageous as long as the patient was on TMP-SMX prophylaxis at the time of stent removal [38].

Urinary tract infections in KT recipients require empiric antibiotic coverage of mainly gram-negative bacteria. In our cohort 89 gram-negative strains of bacteria were isolated compared to 46 gram-positive ones. Gram-negative bacteria were more frequently associated with infections, while gram-positive bacteria were more common in bacteriuria. Empiric antibiotics should therefore always cover gram-negative enterobacteria. There was no resistance to carbapenems in our gram-negative bacteria, which makes them the most attractive choice for critically ill patients. Of the remaining antibiotics Piperacillin / Tazobactam was the substance to which the least gram-negative resistance existed to, at a rate of 15%. Quite frankly, we were surprised how high the bacterial resistance to Ciprofloxacin was in our cohort (29%). Following the 2019 FDA warning against the use of fluoroquinolones in KT recipients, our prescription policy has slowly led to a decrease in Ciprofloxacin use. Given the low susceptibility of uropathogenic bacteria in this cohort the Ciprofloxacin use is still too high to be reasoned. Oral treatment alternatives for empiric treatment of UTIs are highly sought after, but simply did not exist in our study. With a resistance to aminopenicillins in over 50% and a resistance to cephalosporins of nearly 20% we strongly recommend treating urinary tract infections after Kidney Transplantation with empiric intravenous antibiotics according to local resistance patterns. An early step-down to an oral narrow-spectrum antibiotic appears possible once the bacterial strain is identified.

In order to detect ASB and UTI early, our patients undergo weekly screening for urinary pathogens for the first month post-transplant. During this time-frame we identified 59 episodes of ASB (29%) in our cohort which is comparable to other reports [2,8]. Given the lack of randomized controlled trials, most transplant centers treat asymptomatic bacteriuria within the first month after KT [39,40] but other timeframes have been described as well (e.g., treating for 3 months post-op) [41]. At our center we treat ASB within the first month. Recently, more data is becoming available on the treatment of ASB early after transplantation. A meta-analysis from Spain recommended not to treat ASB after the first month post KT [11]. This practice has been backed by two randomized controlled multicenter trials by Origuën et al. and Coussement et al. which both reported no benefits in treating ASB two months after kidney transplant [42,43]. After that time, the prevalence of asymptomatic bacteriuria was reported to be decreasing to about 4% of patients [44]. Thus, further screening is likely unnecessary. Unfortunately, to our knowledge, there are no randomized controlled trials on the treatment of ASB within the first month after KT. Bohn et al. reported on a small cohort of ASBs during the first month post-transplant and found no difference in progression to UTI both when treated or left untreated in their cohort [12]. A potential alternative to screening cultures could be reflex urinary cultures triggered by positive urinary nitrite or a threshold urinary white blood count [45].

The treatment of asymptomatic bacteriuria is the niche for narrow-spectrum antibiotics such as TMP-SMX/Fosfomycin/Nitrofurantoin/Pivmecillinam which are currently not recommended as first line therapy for urinary tract infections in transplant recipients. Nearly all isolated bacteria in our study cohort had a “weak spot” to one of these agents. These agents will grow in popularity given that they are safe and effective even against MRGN bacteria [46,47]. In our study cohort, not a single strain of *E. coli* was resistant to Fosfomycin or Nitrofurantoin, while over 40% were resistant to TMP-SMX. The latter may be an effect of *Pneumocystis* prophylaxis. Keeping in mind that a large proportion of ASB was caused by *Enterococci* however, we need more in-patient studies on the efficacy of the aforementioned antibiotics against *Enterococcus* species [48]. Since there are excellent options available, we strongly advocate against the use of fluoroquinolones in ASB.

While symptomatic patients need to be treated empirically to avoid development of septicemia, we believe it is safe to wait for the final antibiotic resistance testing in ASB and use targeted therapy rather than empiric broad spectrum coverage followed by a step- down approach. This is common practice in pregnant patients but to our knowledge there is yet to be consensus for transplant recipients [9]. The choice of antibiotic for empiric treatment of UTIs must be made with the centers’ urinary tract infection’s spectrum and antibiotic resistance in mind which may vary a lot regionally. Data from antibiotic stewardship teams and hospital hygiene can be helpful, but needs to be observed critically with a regular update. More specifically, the resistance to commonly used antibiotics appears higher in dialysis patients and transplant recipients than in healthy peers. We experienced nearly twice as many resistant strains of *E. coli* in our transplant cohort compared to the regional public. Similarly, while *E. faecalis* was resistant to fluoroquinolones in only 10% of our general population, nearly 90% of *E. faecalis* isolates in our KT recipients were resistant to Ciprofloxacin. Prevention of the development of resistant strains in dialysis patients will be an upcoming challenge for antibiotic stewardship teams worldwide. Only recently have these teams found increasing interest in urologic patients, who are prone to be treated with antibiotics [49]. Antibiotic stewardship data for solid organ transplant recipients and transplant programs appears to be even scarcer [50].

While our MRGN rate of 10% was similar to other German KT centers [51] it was considerably lower than described by Tekkarismaz et al., who reported 41% MRGN urinary tract infections in their cohort in Turkey. Nevertheless 10% was considerably higher than the average of 5% described in a 2017 meta-analysis [52]. The global numbers are increasing and vary considerably even within states and countries.

### Strengths and Limitations

We presented single center data on bacterial isolates from urine samples of over 200 kidney transplant recipients early after kidney transplantation. The retrospective nature of this analysis slightly complicates distinguishing between colonization/bacteriuria and urinary tract infection. Still, we were able to demonstrate distinctly different resistance patterns to commonly used antibiotics in urinary tract infections between our KT recipients and the control group that consisted of all urinary cultures ordered at this tertiary university hospital. Given the uneven sample sizes between the study and control group statistical comparisons were limited to exploratory data. Therefore, multicentric comparative data will be necessary prior to generalization of our data.

## 5. Conclusions

Prevention of infections after surgery as well as rational use of antibiotics is a main goal of antibiotic stewardship programs. Solid organ transplant recipients are at increased risk of fatal infection. This leads to a low threshold for initiating antibiotic treatment when infection is suspected. In our cohort between 207 patients 262 courses of antibiotics were prescribed. Of these, 180 were prescribed antibiotics for suspected urinary tract infection. Lowering these numbers is a team effort of transplant surgeons and physicians accompanied by antibiotic stewardship teams.

## Figures and Tables

**Table 1:** Patient Characteristics.

	No UTI (*n* = 139)	UTI (*n* = 68)	Total	*p*
Gender m (Percentage)	86 (62%)	33 (49%)	119 (57%)	0.068
Living Donor Kidney Transplantation (Percentage)	54 (38%)	19 (30%)	73 (35%)	0.123
Median age in years (Range)	54 (19–82)	60 (18–77)	55 (18–82)	0.034
Mean Body mass index (BMI) in kg/m² (±Standard Deviation (SD))	24.8 (±3.9)	25.6 (±4.1)	25.3 (±4.0)	0.035
Diagnosis Glomerulopathy Polycystic Kidney Disease Hypertension Diabetes Ureteral Disease and Reflux Graft Loss Other	45 (32%) 25 (18%) 9 (6%) 8 (6%) 2 (1%) 12 (9%) 38 (27%)	26 (38%) 14 (21%) 5 (7%) 5 (7%) 3 (4%) 5 (7%) 10 (15%)	71 39 14 13 5 17 48	0.450
Induction Immunosuppression Basiliximab Thymoglobulin Alemtuzumab	88 (63%) 32 (23%) 19 (14%)	51 (75%) 8 (12%) 9 (13%)	139 40 28	0.140
Mean Kidney function in Glomerular Filtration Rate in ml/min (±SD) Discharge 3 Months Follow-Up 12 Months Follow-Up	46.5 (±20) 49.1 (±9) 51.1 (±18)	38.9 (±17) 41.2 (±15) 41.1 (±15)	44.8 (±19) 46.8 (±18) 48.0 (±18)	0.013 0.005 <0.001
Trimetroprime-Sulfamethoxazole Prophylaxis (Percentage)	86 (62%)	31 (46%)	117 (57%)	0.036

This table shows overall patient characteristics and a comparison of both the patients with and without a urinary tract infection (UTI).

**Table 2:** Bacterial isolates from urinary samples of Kidney Transplant recipients.

	UTI	ASB	*n*
*E. coli* *Klebsiella pneumoniae* *Klebsiella oxytoca* *Klebsiella variicola* *Pseudomonas aeruginosa* *Enterobacter cloacae* *Serratia marcescens* *Citrobacter species* *Proteus mirabilis* *Raoultella planticola* *Ureaplasma urealyticum* *Acinetobacter baumanii* *E. faecalis* *E. faecium*	31 13 3 1 2 1 2 2 1 1 6 10	18 1 3 4 2 1 1 1 1 17 13	49 14 6 1 6 3 3 3 1 1 1 1 23 23

This table shows all bacterial strains that were cultured in the cohort and whether a certain bacterial strain was associated with urinary tract infections (UTI) or asymptomatic bacteriuria (ASB). Gram-negative bacteria were more likely to be found in UTIs while gram-positive bacteria were more commonly seen during episodes of ASB.

## Data Availability

The data presented in this study are available on request from the corresponding author. The data are not publicly available du the pseudonymized character of the data.

## Abbreviations

ASB	Asymptomatic Bacteriuria
BMI	Body Mass Index
CG	Control Group
CI	Confidence Interval
FET	Fisher’s Exact Test
GFR	Glomerular Filtration Rate
KT	Kidney Transplantation
MRGN	Multi-resistant Gram-Negative
MWU	Mann-Whitney U-Test
PAP	Perioperative Antibiotic Prophylaxis
POD	Postoperative Day
SD	Standard Deviation
SSI	Surgical Site Infection
TMP-SMX	Trimethoprim-Sulfamethoxazole
UTI	Urinary Tract Infection
Χ²	Chi-Square test
